# Construction of a Banker Plant System via the Host Switch Trait of a Natural Enemy *Aenasius bambawalei*

**DOI:** 10.3390/life13112115

**Published:** 2023-10-25

**Authors:** Juan Zhang, Jun Huang, Yayuan Tang, Yaobin Lu, Zhongshi Zhou

**Affiliations:** 1 Institute of Garden Plants and Flowers, Zhejiang Academy of Agricultural Sciences, Hangzhou 311251, China; juanjuan0031@aliyun.com (J.Z.); yayuan1127@163.com (Y.T.); 2 State Key Laboratory for Managing Biotic and Chemical Threats to the Quality and Safety of Agricultural Products, Key Laboratory of Biotechnology in Plant Protection of MOA of China and Zhejiang Province, Institute of Plant Protection and Microbiology, Zhejiang Academy of Agricultural Sciences, Hangzhou 310021, China; junhuang1981@aliyun.com; 3 College of Forestry and Biotechnology, Zhejiang A & F University, Hangzhou 311300, China; 4 Institute of Plant Protection, Chinease Academy of Agricultural Sciences, Beijing 100193, China

**Keywords:** host switch, synchronization, *Phenacoccus solenopsis*, *Phenacoccus solani*

## Abstract

Understanding the most effective host switch patterns in parasitic wasps, specifically *Aenasius bambawalei* (AB), is crucial for effectively controlling pests like *Penacoccus solenopsis* (PSS). This study aims to elucidate AB’s ideal host switch pattern and assess its utility in maintaining synchronization between AB and PSS, thereby aiding in PSS control. We examined various host switch patterns and cycles to evaluate their impact on AB’s offspring’s parasitism rates and fitness in laboratory conditions. Concurrently, we assessed the fitness of both PSS and AB on tomato plants using different banker plant systems to maintain field synchronization. Results indicate that the three-repeat T1 host switch pattern of PSS-*Penacoccus solani* (PSI)-PSS was the most effective. Additionally, a specific banker plant system, “System B”, which provided succulent plants hosting PSI, was optimal for synchronizing AB and PSS in a summer greenhouse setting. Our findings underscore the importance of employing specific host switch patterns and banker plant systems to effectively control PSS in the field. This research offers foundational data for incorporating a banker plant system into integrated pest management strategies for enhanced PSS control.

## 1. Introduction

The term “host switch of insects” refers to an insect switching from one primary host to an alternative one [1,2,3,4]. Host switching, according to biocontrol theory and practice, may aid in synchronization between biological control agents (BCAs) and their hosts [5]. This synchronization plays a crucial role in population development and pest control of BCAs [6], and that can lead to a reduction in the use of pesticide control. Parasitic wasps, being important BCAs, have been valued for their use as biological pest control in agriculture and forestry [7,8,9,10,11,12]. Research on host switching in parasitic wasps has primarily focused on documenting new host records [13,14], studying the phylogenetic distance between hosts [15], exploring bacterial communities [16] in generalist parasitic wasps [17,18], and so on. However, our understanding of how specialist parasitic wasps utilize their host switch traits to improve their field synchronization with pest hosts remains incomplete [19]. Particularly, the successful establishment of populations of parasitic wasps released into agroecosystems is difficult due to a lack of synchronization, and these populations may be heavily dependent on a variety of means to sustain themselves [20,21,22,23]. As a result, developing strategies to support host switch populations and improve the synchronization between parasitic wasps and their pest hosts could greatly enhance the adoption and effectiveness of host switch techniques in practical applications. 

*Phenacoccus solenopsis* Tinsley (PSS) (Hemiptera: Pseudococcidae) has emerged as a significant invasive pest in China [12,20]. Known for its broad host range [24,25], this polyphagous mealybug poses a threat to both garden plants [26] and economically important crops [27]. High-density populations of PSS can cause extensive damage, resulting in significant economic losses [28]. Traditional pesticide approaches have proven ineffective in controlling PSS due to the biological and physiological traits of PSS [29,30]. Therefore, various research groups have conducted surveys over the past few years to identify the natural enemies of PPS [31,32,33]. Among them, *Aenasius bambawalei* Hayat (AB) (Hymenoptera: Eutrichosomatidae) has been found to be the most predominant and effective parasitic wasp in controlling PSS [12,30,31]. To date, most studies have revealed that AB is the most specific parasitic wasp of PPS. However, the host specificity of AB may vary among different populations of *Phenacoccus* spp. Huang et al. (2020) demonstrated in laboratory trials that AB can successfully switch between PSS and another invasive species called *P. solani* Ferris (PSI) in China [34]. Nevertheless, the multiple host switch behavior of AB and its implications for optimizing the AB population remain poorly understood. Parasitic wasps are fascinating creatures with the remarkable ability to switch between different hosts depending on the season [14]. However, the success of their offspring is influenced by the specific natural enemy hosts encountered in each generation [35,36]. This raises the importance of understanding the effects of multiple host switches in order to develop a robust and high-quality population of parasitic wasps in laboratory settings. Nevertheless, it is crucial to acknowledge that the performance of these wasps can vary in laboratory conditions compared to the natural environment [37]. Hence, it becomes imperative to thoroughly investigate the offspring’s differences before releasing host-switching individuals into the field, ensuring the effective application of the concept of Augmentative Biological Control (ABC). 

Furthermore, achieving sustainable pest control through the release of parasitic wasps in the field requires effective synchronization between the natural enemies and the pest population [38]. In the case of the PSS parasitoid, AB, it is crucial to identify the appropriate host stages for successful parasitism. This includes targeting the second- and third-instar nymphs, as well as female adults of the PSS, while avoiding the first-instar nymphs [31]. Field research has demonstrated that AB can attain high parasitic rates, even reaching 95% or 100% [34]. However, the lack of suitable host stages can hinder the population development of AB and reduce its efficiency in controlling the second-instar or higher PSS, which emerge from the first instar. Introducing alternative host species can increase the probability of synchronization between the biological control agents (BCAs) and their hosts [39]. Nonetheless, it remains unclear how to effectively provide alternative hosts to ensure the fitness and pest control performance of AB, particularly during host switches. One successful method for long-term pest control is the implementation of banker plant systems, which aim to maintain a reproductive population of BCAs within a crop [21,40,41]. These systems have proven effective in controlling scale insects [42] and whiteflies [43,44] in tomato plants. Based on this, we propose the construction of a banker plant system utilizing the host-switching trait of AB. This system would provide an alternative host for the parasitic wasp, facilitating the synchronization of AB and PSS populations and ensuring the successful establishment of the released AB population in agroecosystems.

In the present study, we aim to construct an optimal host switch pattern for AB. Furthermore, we evaluate the effects of releasing host switch AB in conjunction with a suitable banker plant system on the control efficiency of PSS on tomato plants in a greenhouse. Our results will offer valuable insights into the possibility of using host switch traits of a specialist parasitic wasp in the banker plant system and will help consumers estimate the efficiency of this strategy before implementation.

## 2. Materials and Methods

### 2.1. Plants and Insects 

Tomato *Lycopersicon esculentum* Mill (Solanales: Solanaceae) seeds and succulents *Graptopetalum paraguayuense* (Rosales: Crassulaceae) potted plants, which are known to be suitable hosts for the *P. solani* insect, were procured from a reputable supplier located in a bustling market in Hangzhou, China. The potted tomato plants were prepared using the following steps:

Step 1: *L. esculentum* seeds were first soaked in water for a period of 10–12 h. Subsequently, any excess water was carefully removed.

Step 2: The soaked seeds were placed on a moist filter paper, allowing them to sprout (about 7 to 10 days). The seeds were considered ready for the experiment once more than 1/3 of them had sprouted.

Step 3: Once the sprouted seeds reached a height of 20–25 cm, they were transplanted into small pots with a diameter of approximately 15 cm. These pots were designated for use in the experiment.

Throughout the subsequent course of the experiment, both the *L. esculentum* and *G. paraguayuense* potted plants, which had a diameter of 10 cm, were nurtured under standard laboratory conditions. It is important to note that no pathogens, pests, or pesticides were present in any of the plants employed in this study, ensuring the integrity of the results.

The *P. solenopsis* (PSS) and *P. solani* (PSI) strains utilized in this study were initially collected from *Hibiscus mutabilis* Linn (Malvales: Malvaceae) and succulents respectively, in the suburbs of Hangzhou, China. Additionally, the *A. bambawalei* (AB) strain, which was raised on PSS for more than 30 generations, was collected from our laboratory. For the experiment, female adult PSS, PSI, and newly emerged AB, which had mated but had not yet laid eggs, were selected. All experiments were conducted under controlled conditions, with a temperature range of 26–28 °C, relative humidity of 70–80%, and a photoperiod of 16 h light and 8 h dark. This environmental setup was based on the method described by Huang et al. (2020) [34].

Mass rearing is a critical component of biological control programs, serving as the foundation for the applications of biological control agents (BCAs) [45,46,47]. However, the continuous mass rearing of parasitic wasps has been found to have a negative impact on their foraging behavior and overall quality [48]. To mitigate the detrimental effects of long-term mass rearing, one common approach is to introduce parasitic wasps collected from the field, which helps rejuvenate the colony [24]. Another highly recommended method is rearing parasitic wasps on other suitable hosts [39,49]. In this study, we aimed to rejuvenate the colony of the parasitic wasp AB by taking advantage of its ability to switch hosts. Unlike previous field applications that primarily focused on releasing AB populations reared in laboratories on a specific host, PSS, our approach involves releasing AB populations that have undergone host switching. 

### 2.2. Effect of Host Switch Pattern on the Population Traits of Aenasius bambawalei

**Figure 1:**
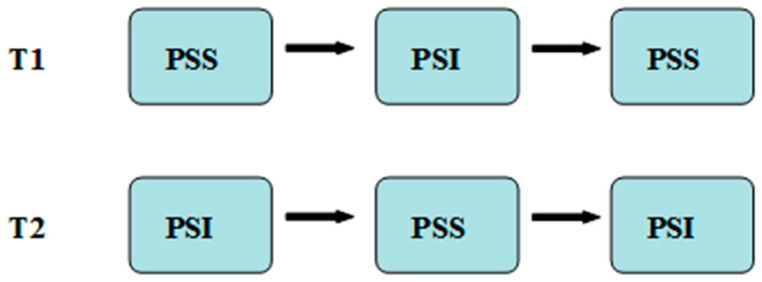
Different host switch patterns of *Aenasius bambawalei*. PSS—*Phenacoccus solenopsis*; PSI—*Phenacoccus solani*.

Previous studies have shown that AB can successfully switch hosts from PSS to PSI after 6 h of adaptation [34]. Thus, according to our preliminary study, two host switch patterns, the T1 host sequence (PSS-PSI-PSS) and T2 host sequence (PSI-PSS-PSI), were selected to clarify the effect of host switch pattern on the population traits of AB. The first pattern, T1, involved rearing AB in PSS. The newly emerged AB from PSS mummies were then transferred to PSI after approximately 14 days of development. These parasitic wasps that emerged from PSI mummies were used to parasitize PSS, resulting in the T1-type AB. Similarly, the T2-type parasitic wasps were obtained by first rearing them on PSI, then PSS, and finally PSI. In the process of host switching, two pairs of newly emerged AB (2 male and 2 female adults) were selected and reared on each host for 48 h and then removed (Figure 1). Among them, the ratio of AB females to PSS and PSI was 2:20 and 2:15, respectively [34]. Then, the newly emerged AB from T1 and T2 patterns were introduced into the cages with *L. esculentum* potted plants for 48 h. Each potted plant was infested with 30 PSS, and the ratio of AB to PSS was 1:30. Additionally, the AB were provided with a daily diet of 20% honey–water solution. Moreover, the parasitic rate, emergence rate, female proportion of offspring, longevity, and the hind tibia length of adult females were observed and counted daily. Each treatment was repeated five times, with the control group consisting of AB continuously reared on PSS. All of the above experiments were carried out in a laboratory at a temperature of 26–28 °C, relative humidity of 70–80%, and photoperiod of L16:D8.

### 2.3. Effects of Different Host Switch Cycles on the Population Structure of Aenasius bambawalei

According to the results in Section 2.2, we selected the most superior host switch pattern and conversion for 1, 2, 3, 4, and 5 cycles, respectively. Similar to the methodology in Section 2.2, AB were introduced into the cages for 48 h, and the ratio of AB to PSS and PSI was 2:20 and 2:15, respectively [34]. After a period of seven days following the removal of AB from the cages, the parasitic rate and ratio of female offspring were determined. AB continuously reared on PSS were used as the control, and each treatment was repeated 3 times. The honey–water solution supplement and experimental conditions were consistent with those described in Section 2.2. 

### 2.4. Construction of the Banker Plant System in Laboratory Conditions

**Figure 2:**
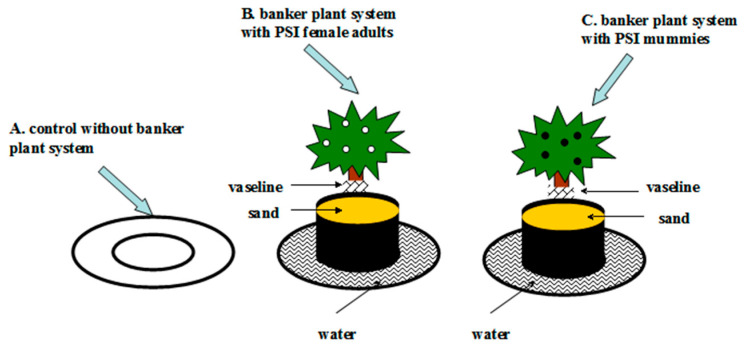
The control without banker plant system (**A**); banker plants with *Phenacoccus solani* female adults (**B**) and with *P. solani* mummies (**C**) in each chamber. To prevent *P. solani* adults from escaping, the stems of plants were coated with Vaseline, the potting soil was covered with sand, and the tray under the potted plant was filled with water.

To fully exploit the advantages of host switches, the banker plant systems were established and used to evaluate their effect on the dynamic of PSS on tomato plants and AB, as well as the parasitic rate of AB. In this experiment, there were two banker plant systems: one with *G. paraguayuense* potted plants housing PSI female adults (referred to as system B), and the other with PSI mummies (referred to as system C) (see Figure 2). In system B, each potted plant accommodated 15 PSI female adults, which were introduced and observed for 1–2 days before further investigation. For system C, a potted plant with 15 PSI female adults was enclosed in a separate cage measuring 40 × 40 × 35 cm, and two cycles of AB subjected to an optimal host switch were added to the cage for 48 h. Afterward, the AB were removed, and the potted plant with PSI was left undisturbed inside the cage for approximately 7–10 days. Subsequently, the unparasitized PSI adult females were gently brushed off, and the potted plant with PSI mummies was employed for subsequent experiments. The control group (referred to as system A) involved potted plants with no PSI female adults or mummies. The supplementation of honey–water solution and the experimental conditions were consistent with those described in Section 2.2. 

### 2.5. Greenhouse Experiment

**Figure 3:**
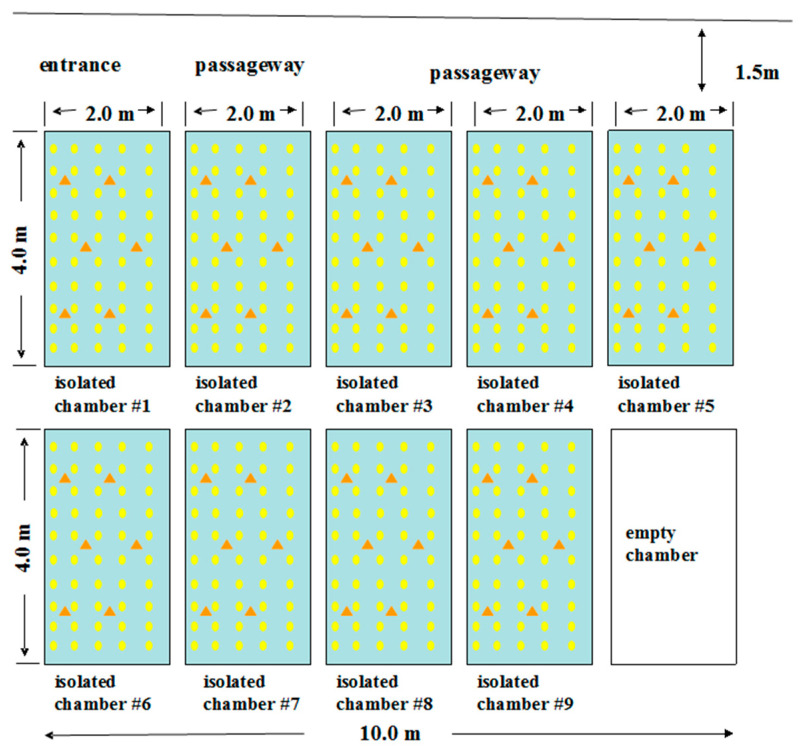
Description of the greenhouse experiment according to Sanchez et al. (2020) and Chen et al. (2022) [42,50]. Yellow dots: position of tomato plants. Orange triangle: position of infested banker plants. In each chamber, only four out of every six orange triangles were used to place the banker plant system.

In the summer of 2021, we conducted an experiment to assess the practical application of *G. paraguayuense*-PSI-AB banker plant systems in greenhouse biocontrol. Our objective was to determine the effectiveness of these systems in controlling pests. The experiment took place in five glass greenhouses measuring 8 m in length, 2 m in width, and 4 m in height, located at the Zhejiang Institute of Garden Plants and Flowers in Zhejiang province, China (118°21′–120°30′ E, 29°11′–30°33′ N). Each greenhouse was divided into two independent chambers measuring 4 m in length and 2 m in width. Plastic membranes were used to separate the chambers and prevent the movement of arthropods between them. In each chamber, we transplanted 50 tomato plants in five rows, with ten plants per row (Figure 3). Thirty randomly selected tomato plants in each chamber were infested with 30 PSS newly emerged adult females. To create the banker plant systems, we transplanted five potted plants of *G. paraguayuense* with either PSI female adults or PSI mummies. These plants were positioned in the six triangle points, as shown in Figure 3, following the methods described by [46,47], with some modifications. In the control chambers, no banker plant system was provided. After an additional two days, we released 10 newly emerged and mated AB female adults (with no host switch) onto each of the ten tomato plants infested with PSS in the control chambers. In the chambers with banker plant systems (B and C), we released AB female adults following an optimal host switch pattern and cycle. Each treatment was replicated in three chambers, randomly selected across the five greenhouses. The insect densities in the greenhouse were chosen based on preliminary surveys conducted by the authors.

The population dynamics of the PSS and the AB on tomato plants were monitored weekly, starting from 7 days after the release of AB and spanning from 9 July to 30 September 2021. At each sampling date, 10 plants per chamber were selected randomly. Then, all PSS individuals (except eggs and the 1st instar) on all plant parts were examined and counted. Finally, in order to obtain one value per greenhouse chamber and per week, we calculated the total number of PSS and mummies per ten tomato plants. The climate inside the greenhouses was subject to seasonal variations, with temperatures ranging between 23 and 28 °C in July, 26 and 29 °C in August, and 28 and 30 °C in September. 

Furthermore, the mummies were removed gently from the plants with insect needles and placed in a Petri dish. Then, approximately 50 mummies were randomly selected on 16 July, 20 August, and 24 September to determine whether offspring sex ratio and size were dependent upon the introduction of the banker plant system. Moreover, the number of female and male offspring was counted, and the length of the hind tibia of 10 newly emerged females was measured under optical electron microscope (Nikon SMZ-10, Nikon Corporation, Tokyo, Japan). Finally, the other AB wasps were introduced into the original greenhouses.

### 2.6. Statistical Analyses 

All statistical analyses were performed with SPSS version 14.0 (SPSS INC., Chicago, IL, USA), and the data were expressed as means ± standard errors. Parasitic rate refers to the percentage of the number of mummies to the total number of mealybugs, while emergence rate refers to the percentage of emerged *A. bambawalei* to the total number of mummies. Meanwhile, the ratio of female offspring refers to the percentage of female offspring to the total number of emerging *A. bambawalei*. The effects of host switch pattern and cycles on the parasitic rates of *A. bambawalei* (laboratory or field population) emergence rate, longevity, offspring female ratio, and hind tibia length were analyzed and compared using one-way ANOVA and post hoc (Fisher LSD) tests, respectively. In order to assess the impact of banker plant systems on the population dynamics of the PSS and the AB on tomato plants, as well as the number of PSS and AB, we conducted a study using three groups: control (no banker plant system), banker plant system B (with PSI female adults), and banker plant system C (with PSI mummies). To analyze the data, a two-way ANOVA was employed to examine the differences between the two banker plant systems and the thirteen different dates. Additionally, multiple comparisons were conducted using Tukey’s test. To compare the variations in offspring sex ratio and size in the field, GLM *t*-tests were utilized and further analyzed using Tukey’s HSD tests for multiple comparisons.

## 3. Results

### 3.1. Effect of Host Switch Pattern on the Parasitism and Fitness of Aenasius bambawalei

**Figure 4:**
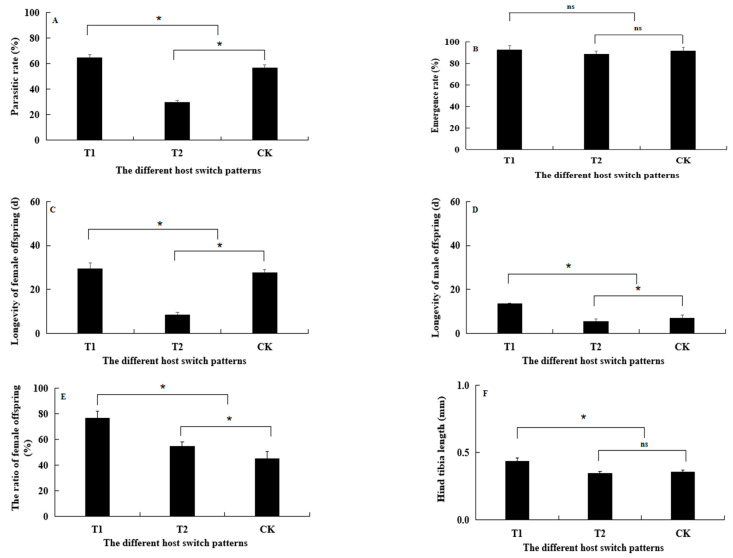
Effect of different host switch on the parasitism and fitness of *Aenasius bambawalei*. Bars labeled with asterisk indicate significant differences among different host switch patterns. (**A**–**F**) represent the parasitic rate, emergence rate, longevity of female and male offspring, the ratio of female offspring, and hind tibia length, respectively. “ns” represent there was no significant difference.

The parasitic rate, the longevity of female and male offspring, and the ratio and size of female offspring showed significant variations depending on the pattern of host switching (Figure 4). In the T1 pattern (PSS-PSI-PSS), all measured parameters, including parasitic rate, longevity of offspring, and ratio and size of female offspring, were superior to those of the control (*p* < 0.05) (Figure 4A,C–F). However, in the T2 pattern (PSI-PSS-PSI), except for the longevity of male offspring (F_(1,8)_ = 2.970, *p* = 1.1231) (Figure 4D) and the ratio of female offspring (F_(1,8)_ = 2.443, *p* = 0.1567) (Figure 4E), all other indices were inferior to those of the control (*p* < 0.05) (Figure 4A,C,D,F). These results suggest that the T1 pattern is more suitable for the parasitism and overall fitness of AB. 

### 3.2. Effect of Host Switch Cycle on the Parasitic Rate and Ratio of Female Offspring

**Figure 5:**
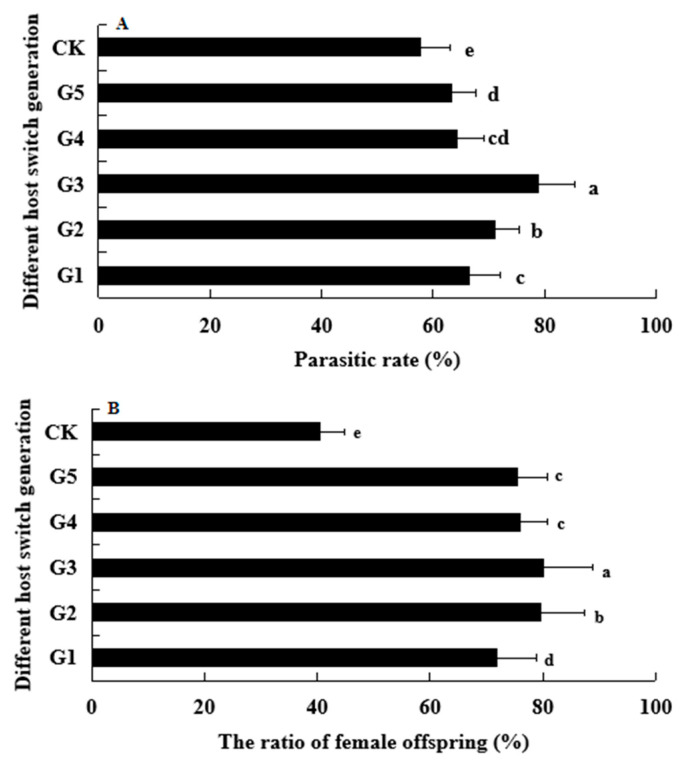
Effect of host switch generation on the parasitic rate (**A**) and ratio of female offspring (**B**). Bars labeled with different lowercase letters indicate significant differences among different host switch generations (*p* < 0.05).

In order to assess the impact of host switch cycles on the population development of the T1 pattern parasitic wasp, two crucial indicators, namely the parasitic rate and the ratio of female offspring, were measured in this study. Remarkably, the parasitic rate (F_(5,12)_ = 13.474, *p* < 0.05) and the ratio of female offspring (F_(5,12)_ = 26.426, *p* < 0.05) exhibited a significant increase across all host switch cycles (Figure 5). Moreover, it was observed that the parasitic rate consistently rose with each subsequent cycle, with statistically significant differences being recorded after three cycles. Specifically, the parasitic rate increased by up to 78.89%, representing a 37.01% increase compared to the control group. Similarly, the ratio of female offspring was up to 80.29%, which was an astonishing 98.39% higher than the control. Subsequently, these two indicators experienced a gradual decline, approaching levels similar to those observed in cycle 1.

### 3.3. Effect of Banker Plant System on the Population Dynamic of P. solenopsis and A. bambawalei

**Figure 6:**
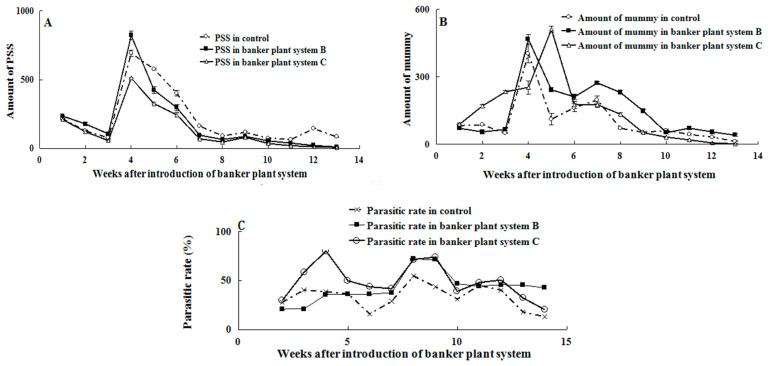
Effect of banker plant system on the amount of mealybug (**A**) and parasitoid (**B**) and parasitic rate (**C**).

To evaluate the effectiveness of using host switch traits of AB to control PSS under field conditions, we implemented a greenhouse experiment using a banker plant system. The results showed significant variations in the number of PSS and AB (F_(24,78)_ =16.315, *p* < 0.05) on tomato plants across different treatments (Figure 6). During the first to fourth week, the number of PSS gradually increased and reached its peak, with some PSS being parasitized, while others laid eggs and the larvae continued to grow. Banker plant system B had the highest number of PSS at this stage. However, the control group had a substantially higher number of PSS on tomato plants compared to banker plant systems B and C (F_(24,78)_ = 61.807, *p* < 0.05) (Figure 6A). Moreover, on the fifth week, the numbers of PSS mummies in banker plant systems B and C were both significantly higher than that in the control (F_(2,6)_ = 19.968, *p* < 0.05). However, there were no significant differences between banker plant systems and the control (F_(2,114)_ = 1.606, *p* = 0.205) on other weeks (Figure 6B).

In terms of the parasitic rate, banker plant systems exhibited much higher rates compared to the control (F_(2,114)_ = 10.102, *p* < 0.05) (Figure 6C). In the third week after release, the parasitic rate in banker plant system C reached up to 80% due to the higher number of AB present, while both system B and the control had rates below 40% at the same stage. The parasitic rate reached its maximum in the seventh to eighth week for all treatments and the control, followed by a decline. However, throughout the experiment, the parasitic rate in banker plant system B consistently remained higher than in system C and the control until the end.

**Figure 7:**
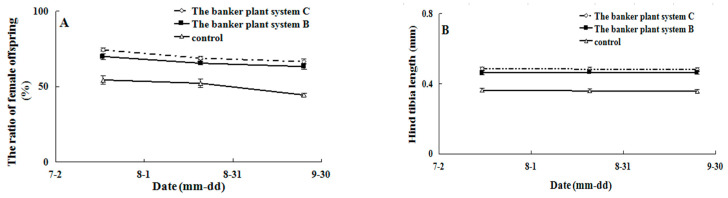
Effect of banker plant system on the ratio of female offspring (**A**) and the hind tibia length (**B**) of *Aenasius bambawalei*.

The influence of banker plant systems on the ratio (F_(2,24)_ = 44.260, *p* < 0.05) and size (F_(2,87)_ = 229.440, *p* < 0.05) of AB female offspring was as predicted, with system C demonstrating greater effectiveness in hind tibia length compared to system B (Figure 7B).

## 4. Discussion

Our results are in accordance with the results of previous studies showing that host switching affects the parasitism and offspring fitness of parasitic wasps significantly [34,50]. In our study, we observed that the parasitic rate of the T1 pattern (PSS-PSI-PSS) was higher compared to the control (PSS-PSS-PSS). Conversely, a significant reduction in the parasitic rate was observed in the T2 type (PSI-PSS-PSI). In our previous study, we set up a host switch as PSI-PSS for 24 h, and the parasitic wasps were reared on PSI as a host for over 10 generations [34]. In the current study, we reared the parasitic wasps on PSS-PSI-PSS for 48 h, and the emerged individuals were subsequently allowed to parasitize PSS for another 48 h. In this case, the parasitic rate in the current study was 1.18 times higher than that in our previous study. These results suggest that the parasitic rate may be influenced by the adaptation generation of the host switch [35,36] and the duration of parasitism [34]. Therefore, we propose that reducing the adaptation time of PSI and extending the parasitic duration of PSS, while continuing to use PSS as the host, can enhance the parasitic rate of the parasitic wasps. In contrast, no difference was observed in the emergence rate in the T2 pattern. In our previous study, the emergence rate in PSI was possibly higher due to the thinner shell of PSI [34]. Whether the ability of the parasitic wasp to break the mummy’s shell or other factors were altered after the host switch remains unknown; thus, further research is needed to investigate the morphological and physiological changes. 

In addition to parasitism, a study by Häckrmann et al. (2007) found that host switching also had a confirmed influence on the fitness of offspring. They suggested that the longevity of parasitic wasp offspring could be largely determined by host size [17]. However, in contrast to previous studies that used the same host in a treatment, in our study, AB underwent a host switch with PSI. PSI, being an inferior host, was smaller in size compared to PSS. Surprisingly, the offspring longevity of the T1 pattern was longer than that of the control group (PSS-PSS-PSS). This suggests that there may be other factors at play that contribute to the longevity of AB offspring, and further research is needed to explore this phenomenon. Furthermore, long-term feeding on mass rearing food negatively affected the foraging behavior and quality of the parasitic wasps [48]. To mitigate these negative effects, it is recommended to rejuvenate the colony by rearing the wasps on other suitable hosts [39]. It would be interesting to investigate whether this rejuvenation process can also contribute to the prolongation of AB offspring’s longevity. The mechanism underlying the differential sex ratio observed in our study is not easily understood. According to theoretical predictions, parasitic wasp mothers are expected to vary their proportion of female offspring based on the available resources, as females often benefit more from a larger body size than males [51]. Therefore, one would expect the parasitic wasp to allocate more daughters to the larger and qualitatively superior host. However, in the T1 and T2 patterns, the opposite was observed. The reasons for this seemingly maladaptive behavior are unclear and may involve constraints during the evaluation of host quality [17] or unknown benefits from the observed behavior. Moreover, the hind tibia length, an important indicator for evaluating the size of parasitic wasps, was larger in the T1 pattern compared to the T2 pattern and the control group. This suggests that the sex ratio of the parasitic wasp population and individual size should be more female-biased in a suitable host switch pattern. Similar to previous studies [35,36], our study also demonstrated that the host switch cycle had a strong and positive effect on population traits of natural enemies. The parasitic rate and the ratio of female offspring reached their maximum in the third cycle and then declined. Additionally, the fitness of the T1 pattern was much higher than that of the T2 pattern, and this difference may be attributed to the cumulative effect of host quality [17]. In conclusion, more controlled laboratory experiments are needed to explore the mechanisms and adaptive explanations behind host switching in order to gain a better understanding of its effects on parasitic wasps.

However, despite the growing body of research on the host switching and preference attributes of parasitic wasps, there is a noticeable gap in the literature regarding the population dynamics and predation parameters of host switching biological control agents (BCAs) under field conditions. This lack of investigation raises concerns, as these parameters play a vital role in the mass rearing and release of BCAs. The issue lies in the fact that most laboratory measurements of BCA attributes are conducted under controlled conditions at a constant temperature, which fails to capture the complexity of field conditions with their fluctuating climatic variations over time and space [37]. Therefore, it is crucial to validate the population dynamics of pests and host switching BCAs, as well as their pest control effects in field settings, particularly when developing integrated pest management strategies. Moreover, the conventional approach of releasing natural enemies comes with several drawbacks. Firstly, natural enemies often struggle to establish successfully in the field, resulting in their limited long-term effectiveness in pest control. Secondly, there is often a lack of synchronization between the presence of BCAs and the abundance of pests, leading to poor pest control outcomes. This necessitates repeated releases of BCAs to sustain control [38]. To tackle these challenges, one potential solution is the implementation of banker plant systems, whereby an alternative host habitat is provided adjacent to the main crop. BCAs can then utilize these banker plants to deliver continuous pest control services within the crop [39]. The concept of banker plants has been extensively developed and researched as a means to boost BCA abundance and enhance pest control efficacy [21,40,41].

Research on the population dynamics of beneficial insects in banker plant systems using alternative prey has garnered significant attention in recent years [42,43,44]. However, there is a lack of reported studies on how to effectively utilize the host switch traits of beneficial insects to construct banker plant systems that ensure the synchronized control of pests. In this study, we developed a banker plant system using the host switch traits of AB in a summer greenhouse. We observed that while the population of PSS on tomato plants was ultimately controlled in all banker plant systems, the dynamics of PSS and the parasitized mummy population varied. In banker plant system B, where only PSI female adults were supplied as alternative hosts for AB, the number of PSS on tomato plants was the highest in the early and mid stages, respectively. This suggested an increase in the number of hosts on the carrier plants, which is not conducive to the effective control of pests by natural enemies on the target crops. However, this assumption was refuted by the fact that the number of PSS on tomato plants was the lowest in the subsequent stages until the end. The continuous growth of first instars of PSS and the availability of more suitable hosts led to a significant increase in the number of PSS parasitized by AB as the host density increased, resulting in more mummies being generated. On the other hand, if the parasitic wasp has strong parasitic abilities and there are too few hosts compared to the number of parasitic wasps in the field during the early and middle stages, it can disrupt the synchronization between natural enemies and pests, ultimately affecting the population development and pest control abilities of beneficial insects. Coincidentally, AB is a parasitic wasp with strong parasitic abilities [34], and the situation in banker plant system C perfectly aligns with what was mentioned earlier. Therefore, it is not surprising that AB has a lower control ability over PSS in the later stages. The implementation of the banker plant system B has been found to be advantageous in achieving synchronization between AB and PSS in a summer greenhouse, as indicated by recent studies. Moreover, our findings have demonstrated that the average rate of parasitism of AB, when it switches hosts, is higher in a summer greenhouse compared to laboratory conditions. This can be explained by the favorable parasitic behavior of AB under moderately high temperatures, implying that temperature plays a significant role in influencing parasitism in natural settings. To gain further insights into the impact of different treatments on the fitness of AB offspring in the field, we conducted a comparative analysis of the proportion and size of female offspring. The results revealed that both the proportion of female offspring and the individual size, particularly the hind tibia length, significantly improved in banker plant systems B and C, as compared to the control group. This provides evidence that host switching can effectively enhance the fitness of the parasitic wasp population. Although the proportion and size of female offspring observed in our study were slightly lower than those observed under controlled conditions, the efficacy of AB control in practical applications suggests the potential utility of employing banker plant system B in field settings. It is worth noting that PSS and PSI belong to the same genus and share similar host profiles. When using PSI as an alternative prey, precautions should be taken to prevent PSS and PSI from harming each other between the tomato and banker plants [34]. In this study, we prevented mealybugs from escaping by coating the succulent stalks with Vaseline, covering the potting soil with sand, and irrigating the pots with water according to the method of Zang et al. (2020) [40].

## 5. Conclusions

In this study, we conducted an in-depth exploration of the host switch pattern and appropriate host switching of AB. Our primary focus was on developing a banker plant system that incorporates succulent plants and PSI female adults (or PSI mummies) to assess its effectiveness in real field conditions. The results revealed that treatment B, which utilized the host switch traits of AB, not only effectively controlled PSS on tomato plants but also maintained synchronization between AB and PSS on tomato plants. These findings are of the utmost importance in elucidating the host switch phenomenon of AB, and hold significant implications for the establishment of banker plant systems that harness the host switch traits of natural enemies to control invasive pests. The successful implementation of such systems has the potential to revolutionize pest control strategies. However, it should be noted that further research is required to uncover the underlying mechanisms of AB’s host switching and to fully maximize its potential in future applications.

## Data Availability

Data are available on request with the approval of the Zhejiang Academy of Agricultural Sciences.
